# Quantitative Evaluation of the Reticuloendothelial System Function with Dynamic MRI

**DOI:** 10.1371/journal.pone.0103576

**Published:** 2014-08-04

**Authors:** Ting Liu, Hoon Choi, Rong Zhou, I-Wei Chen

**Affiliations:** 1 Department of Radiology, University of Pennsylvania, Philadelphia, Pennsylvania, United States of America; 2 Department of Materials Science and Engineering, University of Pennsylvania, Philadelphia, Pennsylvania, United States of America; Johns Hopkins University, United States of America

## Abstract

**Purpose:**

To evaluate the reticuloendothelial system (RES) function by real-time imaging blood clearance as well as hepatic uptake of superparamagnetic iron oxide nanoparticle (SPIO) using dynamic magnetic resonance imaging (MRI) with two-compartment pharmacokinetic modeling.

**Materials and Methods:**

Kinetics of blood clearance and hepatic accumulation were recorded in young adult male 01b74 athymic nude mice by dynamic T2* weighted MRI after the injection of different doses of SPIO nanoparticles (0.5, 3 or 10 mg Fe/kg). Association parameter, K_in_, dissociation parameter, K_out_, and elimination constant, K_e_, derived from dynamic data with two-compartment model, were used to describe active binding to Kupffer cells and extrahepatic clearance. The clodrosome and liposome were utilized to deplete macrophages and block the RES function to evaluate the capability of the kinetic parameters for investigation of macrophage function and density.

**Results:**

The two-compartment model provided a good description for all data and showed a low sum squared residual for all mice (0.27±0.03). A lower K_in_, a lower K_out_ and a lower K_e_ were found after clodrosome treatment, whereas a lower K_in_, a higher K_out_ and a lower K_e_ were observed after liposome treatment in comparison to saline treatment (P<0.005).

**Conclusion:**

Dynamic SPIO-enhanced MR imaging with two-compartment modeling can provide information on RES function on both a cell number and receptor function level.

## Introduction

The reticuloendothelial system (RES) plays an important role in inflammatory responses, antigen presentation, and immunoregulatory networks [1], [2], depression of its function is likely to weaken the host defense. Since the hepatic RES contains the largest number of fixed tissue macrophages (Kupffer cells) and accounts for more than 80% of the entire RES [2], [3], impairment of the liver has a major influence on RES function. Bacterial infections is the most common complication of chronic liver injury and after partial hepatectomy [4]–[6]; therefore the precise estimation of RES function is very important. Bloodstream clearance of radiolabelled particles has been used to measure the function of the reticuloendothelial system [7]–[10]. This technique involves injecting a dose of radiolabelled particles intravenously, withdrawing venous blood samples at time intervals and calculating the clearance rate from the measurement of the radioactivity of each sample. However, this approach is invasive, involving exposure of the subjects to radiation, and is unlikely to be implied in the clinic. Thus, a more efficient, accurate, and safe modality needs to be developed for measurement of the reticuloendothelial phagocytic capacity.

Superparamagnetic iron oxide nanoparticles (SPIO) have been used as a liver-specific MRI contrast agent for detecting hepatocellular carcinoma [11], [12]. This technique relies on the ability of kupffer cells (KC) to uptake SPIO nanocrystals, which leads to reduced signal intensity (SI) on T2 MRI. Because the phagocytic activity of KC decreases as liver function deteriorates in chronic liver disease, SPIO mediated hepatic SNR change is reduced in patients with impairment of liver function on T2 and T2*-weighted MRI. However, single pre- and post-contrast images can only provide information on SPIO uptake at a certain time-point; thus, it remains controversial whether the decrease in liver uptake of SPIO is due to reduced kupffer cell density or impairment of individual macrophage function. Furthermore, the change of MR signal intensity of tissue after administration of SPIO nanocrystals are caused not only by SPIO organ uptake but also by subsequent SPIO enrichment within cellular structures which can dramatically increase r2* relaxivity and prevent the accurate quantification of SPIO uptake kinetics [13], [14]. Several studies have shown that it is possible to adjust the relaxivities to the static dephasing regime by embedding SPIO within the lipid core of micelles, allowing accurate quantification of SPIO uptake kinetics [15], [16]. The purpose of this study was to quantify the phagocytic behavior of RES system by real-time imaging blood clearance as well as hepatic uptake of lipid-based SPIO nanoparticles using dynamic MRI with two-compartment pharmacokinetic modeling. The association parameter, K_in_, dissociation parameter, K_out_, derived from dynamic MRI data, were used to investigate the SPIO nanoparticle active binding to Kupffer cells, which may offer insight into kupffer cell function and density.

Recently, the encapsulation of the bisphoaphonate clodronate into liposomes has been developed as an efficient reagent for the selective depletion of macrophages in RES and successfully applied in several immunological studies [17], [18]. Liposomal blockade of RES has been showed its ability to block RES uptake of second dose of particles by i.v. administration of a large dose of liposomes [19], [20]. This strategy is based on blocking the interaction between the second dose of particles and the receptors on the macrophage cell surface. We therefore utilized the clodrosome-mediated macrophage “suicide” approach to reduce the number of macrophages in RES system and the ability of liposome on the RES saturation to evaluate the capability of the dynamic SPIO-enhanced MRI with two-compartment pharmacokinetic modeling for investigation of Kupffer cell function and density.

## Materials and Methods

### 1 Contrast agents

Fe(III) acetylacetonate (2 mmol) was added to phenyl ether (20 mL) with 1,2-hexadecanediol (10 mmol) and cholic acid (12 mmol) under N_2_, then heated to 265°C to form iron oxide (IO) coated with cholesterol acid (chol-IO). 80% of 1-palmitoyl-2-oleoyl-sn-glycero-3-phosphocholine (PL, Avanti Polar Lipids), 10% of 1,2-dipalmitoyl-sn-glycero-3-phosphoethanolamine-N-[methoxy(polyethylene glycol)-2000] (PEG-PL, Avanti Polar Lipids) and 10% of 3F in methanol were added to chol-IO or Qdot 800 ITK organic quantum dots in chloroform and dried in N_2_ at room temperature to form thin films. Phosphate buffer solution (PBS) was added to the dried film, followed by sonication at room temperature. The NPs were collected in a centrifuge at 15,000 rpm (Optima L- 90K ultracentrifuge, Beckman Coulter). The shell-to-core weight ratio was 15∶1. The Qdot 800 ITK organic quantum dots with emission wavelength of 800 nm (QD800) were purchased from Invitrogen Corporation, CA. The size and zeta potential of NP were measured by a particle size analyzer (Zetasizer 3000HS, Malvern). To verify the Fe concentration, 20 mL of IO-containing sample was dissolved in 980 mL of 5 M hydrochloric acid and the absorbance at 410 nm was measured by an UV-VIS spectrometer (Evolution 201, Thermo Scientific) with reference to calibration standards. The relaxivities of SPIO nanoparticle were measured at 9.4 T and 37°C.

To evaluate the r2* relaxivity of intracellular SPIO nanoparticles, Raw 264.7 cells were incubated in presence of SPIO nanoparticles at 37°C. Iron concentrations in culture medium (DMEM) were adjusted to 0.1, 0.5, 1.0, and 3.7 mg Fe/mL. The concentration of cells in the culture medium was 2×10^6^ cells per milliliter. After 1 hr, cells were collected and suspensioned in agarose gel (1%) for r2* relaxivity measurement. The Fe concentrations of cells were measured using same procedure as free SPIO nanoparticles. Measurements were done in triplicate.

To investigate macrophage cell viability after SPIO nanoparticle uptake, Raw 264.7 cells were incubated in the absence (PBS treatment) or presence of SPIO nanoparticles at 37°C. Iron concentrations in culture medium were adjusted to 0.5, 1.0 and 2.0 mg Fe/mL. The concentration of cells in the culture medium was 2×10^6^ cells per milliliter. After 10, 20 and 30 mins, Raw 264.7 cells were processed for viability measurement or Prussian blue staining. Cell viability was determined by using trypan blue exclusion assays for all samples. Dead cells were counted with the microscope in a total area of 25 mm^2^ (approximately 150 cells) by using a calibrated counting chamber. For Prussian blue staining, cells were fixed in acetone and incubated with a 1∶1 vol/vol mixture of 2% potassium ferrous cyanide (kaliumhexacyanoferrat [II]) and 2N HCl for 30 minutes. The presence of iron oxides was quantitatively assessed with a microscope by estimating iron-positive cells from a total of approximately 150 cells. Measurements were done in triplicate.

### 2 Animals

Young adult male 01b74 athymic nude mice (n = 24) were used in the present study. To establish pharmacokinetic model for evaluation of RES function, 10 animals received an intravenous bolus dose of SPIO nanoparticles in concentrations of 0.5, 3 or 10 mg Fe/kg during dynamic MRI. For macrophage depletion study, 4 animals were intravenously treated with clodrosome liposome (Encapsula NanoSciences) 50 mg/kg once daily for 2 days before dynamic MRI. For RES blockade, 4 animals were intravenously treated with 376 mg L-α-Phosphatidylcholine/kg encapsulated liposome (Encapsula NanoSciences) 1.5 hrs before dynamic MRI. Animals were anesthetized during experimentation with 1 to 1.5% isoflurane by nosecone. To investigate the phagocytic behavior of RES system, 8 mice in two animal models were received an intravenous bolus dose of SPIO nanoparticles in the concentration of 10 mg Fe/kg during dynamic MRI. After MRI, mice were sacrificed by cervical dislocation. Liver and spleen were excised and subjected to immunostaining and Prussian blue staining for investigation. The experimental design was approved by the University of Pennsylvania Committee on Use and Care of Animals. The protocol was approved by the Committee on the Ethics of Animal Experiments of the University of Pennsylvania (Permit Number: 804748).

### 3 In vivo MRI

The mice were examined in a 9.4 T/31 cm horizontal bore magnet with a 21 cm ID gauss/cm and a 12 cm ID gauss/cm gradient tube and interfaced to a Varian Direct Drive console (Varian, Palo Alto, CA, USA). A 35 mm ID volume coil was used to all MR images. During imaging, the free-breathing mice were sedated by 1–1.5% isoflurane mixed with oxygen, and the core temperature, electrocardiogram (ECG) and respiration were monitored (SA Inc, Stony Brook, NY). The core temperature was maintained at 37°C by directing warm air into the bore, while the respiration signals were used to gate the data acquisition to minimize motion interference.

Dynamic T2*-weighted MR imaging was performed using a two-dimensional T2-weighted gradient-echo sequence (TR/TE  = 7/3.5 ms, flip angle 10^°^, matrix of 128×128, a 30×30 mm field of view). Transversal images of the abdomen containing the liver, the stomach, and major abdominal vessels (aorta, inferior vena cava and portal vein) were consecutively taken every 3.2 s. This sequence was applied continuously 440 measurements. Injection of SPIO nanoparticles was started at measurement 34, directly followed by a 100 µl saline flush. The SPIO nanoparticle was administered manually in approximately 25 s. In consequence, the kinetics of distribution of SPIO nanoparticles can then be imaged *in vivo* by measuring dynamic time series.

### 4 Pharmacokinetic Modeling

**Figure 1:**
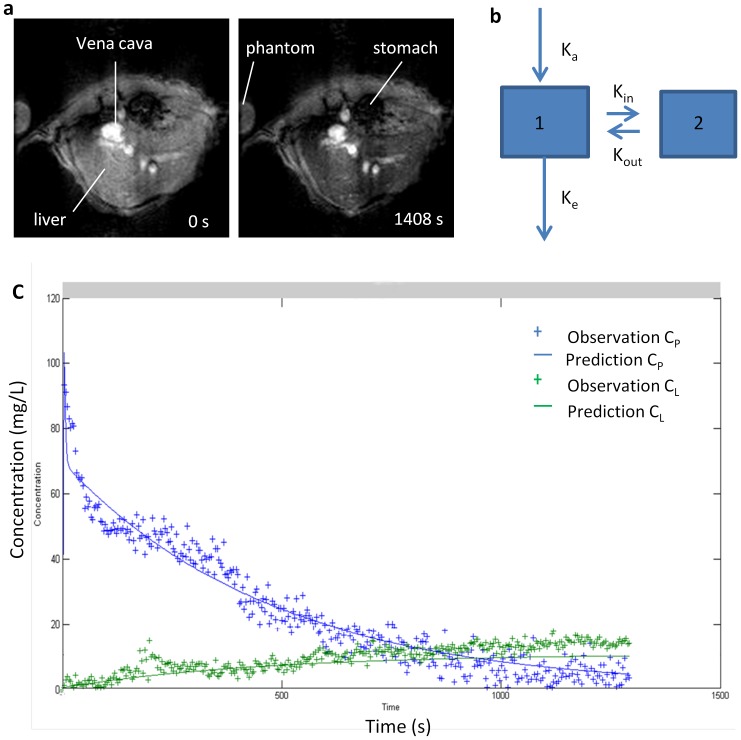
a. Transversal T2* MR images of liver before (left) and 20 min (right) after intravenous injection of SPIO-NP in the concentration of 10 mg Fe/kg. The negative contrast indicates the accumulation of SPIO-NP in liver. b. Schematic of the two compartment model used to describe the data. Compartment 1 and 2 are blood and liver, respectively. The model is entirely determined by the values of the four constants, K_a_ (s^−1^), K_e_ (s^−1^), K_in_ (s^−1^), and k_out_ (s^−1^). c. Simultaneous pharmacokinetic fitting of SPIO nanoparticle in the blood and liver. Individual measurements (crosses) and the determined fit (solid line) are shown.

Since the hepatic RES contains the largest number of fixed tissue macrophages (Kupffer cells) and accounts for more than 80% of the entire RES [2], [3], the uptake of particle by Kupffer cells has a major influence on the concentration of particles in bloodstream. Furthermore, biodistribution of SPIO nanoparticles showed the clearance from blood was accomplished by liver and extrahepatic RES tissue, and most of SPIO nanoparticles were accumulated in liver. Thus, a two-compartment pharmacokinetic model was developed to describe the behavior of the SPIO nanoparticles *in vivo* (Fig. 1 b). Compartment 1 represents the bulk distribution of SPIO nanoparticles in bloodstream with distribution volume V_p_. An absorption constant, K_a_, is used to describe administration of SPIO nanoparticles into blood compartment, which represents the relationship between the injected dose and the dose of SPIO nanoparticles in the blood. In one hand K_a_ can be influenced by different mouse condition and duration of injection in the experiment, in another hand it can be governed by r2* relaxivity of SPIO nanoparticles in the blood. It should be approximately 1 for the intravenous bolus injection, when r2* relaxivity does not change during the measurement process. Therefore, whether the dynamic data were selected for further analysis was based on K_a_ values. An elimination constant, k_e_, is used to describe clearance of SPIO nanoparticles by extrahepatic RES. The liver is assumed to be the compartment 2 where macrophage uptake can occur. K_in_ and K_out_, are lumped parameters that are used to describe both passive transfer and active binding. Particle binding to the macrophages involves two reversed processes, which are association and dissociation. K_in_ (s^−1^) is used to describe the association process, and dissociation is characterized by the constant K_out_ (s^−1^). To adequately analyze the effect of active binding, distribution volume of liver compartment V_L_ was fixed at the reasonable value (1×10^−3^ L/kg). The bolus dose administered for each mouse was 0.5, 3 or 10 mg Fe/kg body.

The ordinary differential equations that govern this model are:
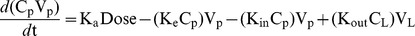


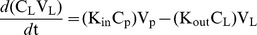



Where C_p_ and C_L_ are the concentrations of SPIO nanoparticles in blood and liver, respectively. The other variables have already been identified above.

### 5 Data analysis

The dynamic data were processed with the computer program ImageJ (National Institutes of Health, NIH). Regions of interest (ROIs) were placed in the liver and the vena cava and intensities per pixel of every single image in a time series S(t) were exported and normalized to the average intensity S_0_ contrast before SPIO nanoparticles injection. The liver ROI was drawn precisely to include all lobes while excluding larger vessels. The change of the relaxation rate R2*  =  (1/T2*) can be calculated by ΔR2*  =  −In[S(t)/S_0_]/TE [21]. This measure scales linearly with the concentration of SPIO nanoparticles ΔC = ΔR2*/r2* when r2* relaxivity does not change during the measurement process.

Modeling, simulation and analysis were done in Matlab (v7.12.0 R2011a, Mathworks) using the SimBiology toolbox and the simulation were run on a laptop PC. All events were modeled using mass action kinetics. All first-order rates have units of 1/second, and all concentrations are defined in mg/L. Simulation was performed using the sundials solver routine with an absolute tolerance of 10^−20^ and a relative tolerance of 10^−5^. The estimation of parameters was treated as a nonlinear optimization problem. Parameter estimation was done using a combination of fitting by hand and local minimization functions from the Matlab Optimization toolbox. Specifically, initial values of parameters were adjusted by hand to get an approximate fit to the data. The values obtained were then used as initial guesses for the simultaneous fitting, which is performed by minimizing the sum of squared residuals.

### 6 Histological analysis

Tissues (liver and spleen) were excised upon euthanasia, embedded in OCT medium and snap frozen. Twenty micrometer sections were then cut and fixed in 10% paraformaldehyde, followed by either Prussian blue staining for detection of SPIO nanoparticles or immunostaining of macrophages. For Prussian blue staining, tissue sections were incubated for 1 hr in dark with 10% potassium ferrocyanide dissolved in 20% hydrochloric acid; they were finally counterstained with nuclear fast red. For immunostaining of macrophages, CD 68 antibody (rat-anti-mouse, AbD Serotec, USA) was used as the primary antibody. Primary antibody was detected by sequential incubation with secondary antibody (goat anti-rat IgG conjugated to FITC, Life Technologies, NY, USA). Images were captured and processed with an epifluorescence microscope (Nikon E600 Upright Microscope, NY, USA) and ImageJ software (NIH, USA). The macrophage densities were calculated by the area of macrophages per field area of digital photomicrographs. The numbers of SPIO nanoparticles were quantified by the ratio of the area of blue spots to field area of digital photomicrographs.

### 7 *Ex vivo* optical imaging

To investigate the tissue distributions of the nanoparticles, 6 mice received an i.v. injection of nanoparticles at a dose of 10 mg Fe/kg or saline, and were sacrificed 20 mins post-injection. The major organs including the liver, lung, spleen, kidney, brain, and heart were dissected from the mice, and their fluorescence intensities were determined using a IVIS Spectrum Pre-clinical *In Vivo* Imaging System (PerkinElmer, USA) equipped with a QD800 band pass filter (excitation/emission 675/800 nm). The fluorescence intensities of organs treated with nanoparticles were averaged by the area of organs in the image and normalized with that in saline group.

### 8 Statistical analysis

Statistical analysis was performed by using SPSS software (version 17.0J for windows; SPSS, Chicago, IL, USA). Numeric data are reported as the mean ± standard deviation. P<0.05 was considered statistically significant.

## Results

### 1 SPIO Nanoparticle characterization

SPIO nanoparticles were nominally 25 nm in diameter with 8 nm core. The transversal relaxivities of SPIO nanoparticles were 210 mM^−1^s^−1^ for r2 and 320 mM ^−1^s^−1^ for r2* at 9.4T and 37°C.

**Figure 2:**
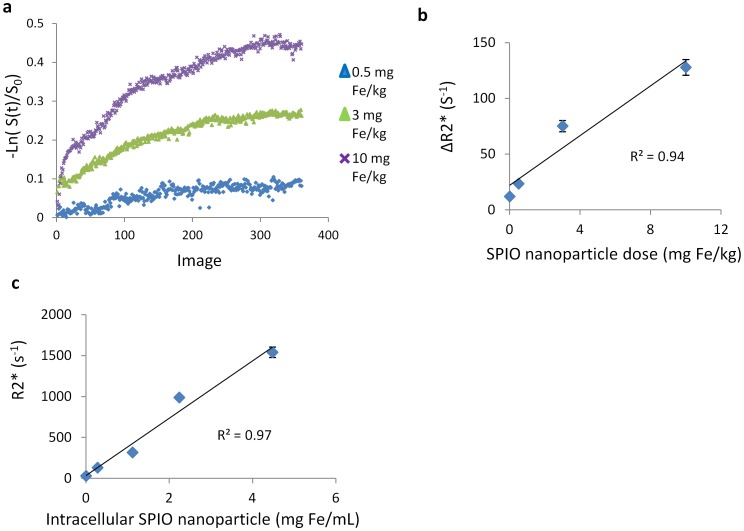
a. Time course of hepatic SPIO-NP uptake kinetics after injection of different doses of SPIO nanoparticle. b. A linear association between the iron dose and the change in the relaxation rate (ΔR2*) in the liver 20 mins after injection of SPIO nanoparticle was observed (R^2^ = 0.94, P = 0.04, n = 3). c. Relaxometry of cell phantom. A linear increase of R2* values of cell phantom with increasing Fe concentration was observed (R^2^ = 0.97, P = 0.03, n = 3).

Figure 2 panel a is the time courses of hepatic uptake kinetics after injection of different doses of SPIO nanoparticles (0.5, 3, and 10 mg Fe/kg). A linear association between the iron dose and the change in the relaxation rate (ΔR2*) in the liver 20 mins after injection of SPIO nanoparticle was observed (R^2^ = 0.94, P = 0.04) (Fig. 2 b). Five cell phantoms with different Fe concentrations (0, 0.016, 0.063, 0.125, 0.250 mg Fe/mL) were used to determine r2* relaxivity of intracellular SPIO nanoparticles. A linear increase of R2* values of cell phantom with increasing SPIO concentration was observed (R^2^ = 0.97, P = 0.03), therefore allows to quantify bloodstream clearance and hepatic uptake kinetics (Fig. 2 c). The r2* relaxivity of cell phantom was ∼350 mM^−1^s^−1^, which was in good agreement with free SPIO nanoparticle (320 mM^−1^s^−1^).

**Figure 3:**
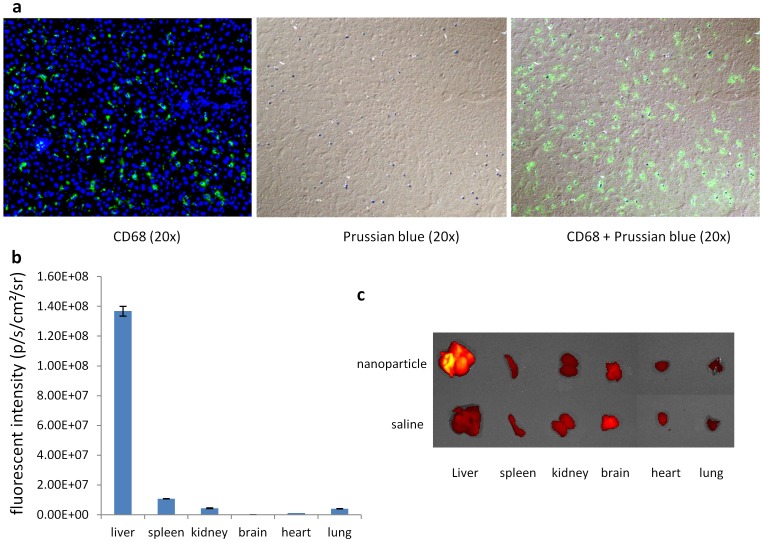
a. Prussian blue staining and fluorescent double-immunohistochemistry with CD68 antibody of liver treated with SPIO nanoparticle in the concentration of 10 mg Fe/kg. It showed that the blue dye was localized in kupffer cells. b. Biodistribution was determined 20 min after intravenous injection of nanoparticle (n = 3). A heavy accumulation of nanoparticle was found in liver. c. Representative *ex vivo* NIR images.

We evaluated cell uptake of SPIO nanoparticle using Prussian blue staining and fluorescent double-immunohistochemistry with CD68 antibody in liver section, it showed that the blue dyes were localized in kupffer cells (Fig. 3 a). These data indicated that SPIO nanoparticles were exclusively phagocytosed by the kupffer cells and it is possible to use SPIO nanoparticles enhanced MRI to evaluate RES phagocytosis function. We further investigated the biodistribution of SPIO nanoparticles by embedding QD into nanoparticles using *ex vivo* optical imaging, it showed that the nanoparticles were heavily accumulated in liver (Fig. 3 b, c).

We studied the cell viability after SPIO nanoparticle uptake. No decrease in cell viability was observed after SPIO nanoparticle incubation up to a concentration of 2.0 mg Fe/mL (Table 1). Increasing the incubation time resulted in an increase in iron-positive cells in 30 mins (Table 2).

**Table 1:** Cell viability of Raw 264.7 cells after SPIO nanoparticle uptake.

	0 mg Fe/mL	0.5 mg Fe/mL	1 mg Fe/mL	2 mg Fe/mL
10 mins	86±2%	87±2%	85±2%	86±1%
20 mins	86±2%	91±3%	87±2%	84±2%
30 mins	85±2%	86±1%	84±1%	82±2%

**Table 2:** Iron-positive Raw 264.7 cells after SPIO nanoparticle incubation.

	0.5 mg Fe/mL	1 mg Fe/mL	2 mg Fe/mL
10 mins	22±1%	27±3%	29±2%
20 mins	43±4%*	53±3%*	59±4%*
30 mins	68±5%*	73±4%*	74±7%*

* P<0.05 versus previous time point. n = 3.

### 2 Blood and liver time curves

Rapid in vivo MR imaging (3.2 s per frame) was performed on a transverse imaging slice through the liver and vena cava to capture simultaneously the passage of SPIO nanoparticles from the blood compartment and accumulation in the liver. The time curve from vena cava exhibits a sharp increase followed a typical bi-exponential decay. Meanwhile, upon injection of SPIO nanoparticles, a continuous rise in the liver time curve was observed, indicative of SPIO nanoparticle accumulation (Fig. 1 c).

### 3 Pharmacokinetic modeling

**Figure 4:**
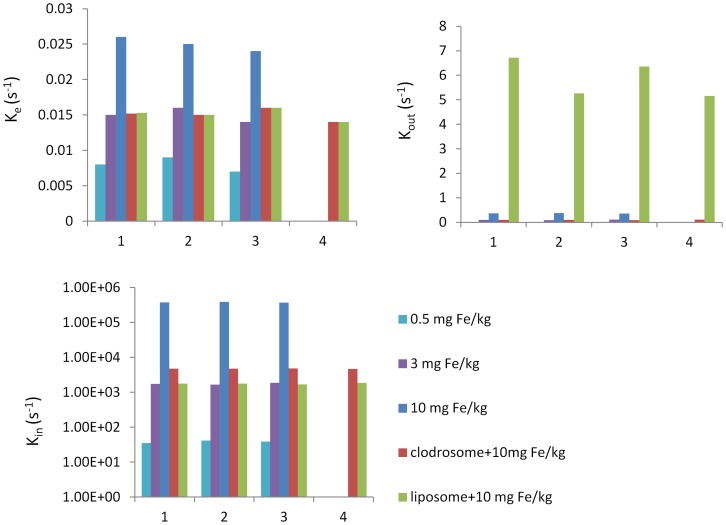
Bar charts showing the distribution of fitted parameters from the simultaneous fitting using the two compartment model. Numbers on x-axis are animal numbers.

Based on biodistribution of SPIO nanoparticle, a two-compartment mathematical model was defined, comprising the plasma and hepatic RES compartments to evaluate the RES system function. The parameter k_in_ and k_out_ were used to describe the SPIO nanoparticle associated to and dissociated from the macrophage, and elimination constant, k_e_, was used to describe clearance of SPIO nanoparticle from blood compartment by the extrahepatic RES. Figure 1 shows the example plots illustrating the results of simultaneous fitting of both the blood and liver compartment for an individual normal mouse treated with a dose of SPIO nanoparticles in the concentration of 10 mg Fe/kg. One mouse in 10 mg Fe/kg group was removed from the individual fit due to the high K_a_ value. The two-compartment model provided a good description for all data and showed a low sum squared residual for all mice (0.27±0.03). Figure 4 shows the distribution of fitted parameters from the simultaneous fitting using the two compartment model. A lower K_in_, a lower K_out_ and a lower K_e_ for low dose of SPIO nanoparticles were found in comparison to high dose (p<0.005) (Table 3).

**Table 3:** Comparison of fitted parameters from two-compartment model.

	0.5 mg Fe/kg	3 mg Fe/kg	10 mg Fe/kg	Clodrosome +10 mg Fe/kg	liposome +10 mg Fe/kg
K_in (_s^−1^_)_	38.527±3.512**	1750.480±100**	378027.6±9504.385	4750.368±83.616 **	1770.913±73.476**
K_out__(_s^−1^_)_	0.014±0.001**	0.098±0.01**	0.365±0.01	0.128±0.056 **	5.875±0.783**
K_e__(_s^−1^_)_	0.008±0.001**	0.015±0.001**	0.025±0.001	0.015±0.001**	0.015±0.001**

**p<0.005 compared with 10 mg Fe/kg.

### 4 Macrophage depletion and functional blockade

**Figure 5:**
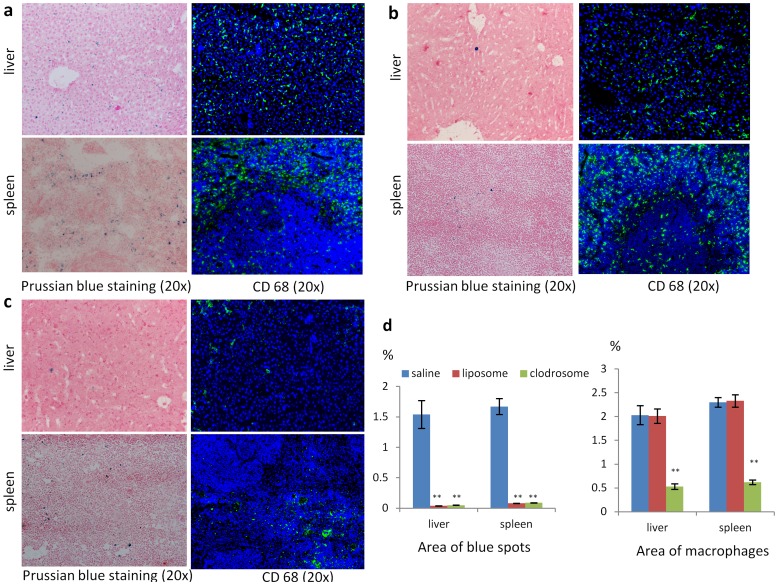
Prussian blue staining and CD68 immunostaing of liver and spleen in saline (a), liposome (b) and clodrosome (c) group. The blue dots on Prussian blue staining are the liver-accumulated SPIO nanoparticles; the green fluorescence on fluorescence images is macrophage. d. The area occupied by kupffer cells and SPIO nanoparticles estimated by imaging analysis in liver and spleen. (**p<0.005 compared with saline group. n = 4).

After dynamic MRI, mice were sacrificed and livers and spleens were collected for Prussian blue staining and immunostaining. To quantify the number of macrophages and SPIO nanoparticles, the area occupied by macrophages and blue spots was estimated by image analysis. A significant decrease in the number of CD68 positive cells in livers and spleens treated with clodrosome was found (0.53±0.06%, 0.62±0.05%, P<0.005), whereas no difference in macrophage number in livers and spleens was observed between liposome treatment (2.01±0.15%, 2.33±0.13%) and saline treatment (2.03±0.21%, 2.31±0.12%) (Fig. 5). Few of blue dye were found in liver and spleen treated with clodrosome (0.05±0.00%, 0.09±0.00%) or liposome (0.04±0.00%, 0.08±0.00%) compared to saline (1.54±0.23%, 1.67±0.13%) treatment at same dose of SPIO nanoparticles (P<0.005, Fig. 5).

A lower K_in_, a lower K_out_ and a lower K_e_ were found after clodrosome treatment, whereas a lower K_in_, a higher K_out_ and a lower K_e_ were observed after liposome treatment in comparison to saline treatment (P<0.005, Fig. 4, Table 3).

## Discussion

Phagocytic activity of macrophages in RES has been reported to be reduced in chronic hepatic disease [22], which is dependent on both kupffer cell tissue density and on individual kupffer cell function. Despite numerous investigations of RES function in chronic hepatic injury [23]–[26], to our knowledge, no clinical method has been reported to distinguish kupffer cell tissue density from individual kupffer cell function in the hepatic RES. Although the number of kupffer cells can be estimated with immunohistology staining, it is difficult to evaluate kupffer cell function either *ex vivo* or *in vivo*. Bloodstream clearance of radiolabelled particles has been used to measure the function of reticuloendothelial system [8], [10]. However, this approach is invasive, involving exposure of the subjects to radiation, and is unlikely to be implied in the clinic. Therefore, a noninvasive, accurate and quantitative method for assessment of macrophage function and density would be desirable. Hepatic signal intensity change at SPIO enhanced MRI is mediated by phagocytic activity [27]–[29], which has great potential to be used to quantitatively evaluate the RES function. Furthermore, kinetics of disappearance from the bloodstream and hepatic accumulation of intravenously injected SPIO nanoparticles may provide a better understanding of the pathological process of impaired phagocytosis in chronic hepatic injury. Since macrophage plays an important role in inflammatory responses, which is mediated by multiple receptors (e.g., complement receptors, chemokine or interleukin receptors, and the Fc receptor) [30], [31]. Several studies have demonstrated that the impairment of macrophage receptor function contributed to macrophage dysfunction in patients with cirrhosis [32]. Thus, association parameter, K_in_, dissociation parameter, K_out_, derived from dynamic MRI data, were used to investigate the functional changes of macrophage receptors in present study, which may offer insight into KC function and density and may help for the correct staging of chronic liver disease. However, the change of MR signal intensity of tissue after administration of SPIO nanocrystals are caused not only by SPIO organ uptake but also by subsequent SPIO enrichment within cellular structures which can dramatically increase r2* relaxivity and prevent the accurate quantification of SPIO uptake kinetics [13], [14]. Embedding SPIO within the lipid core of micelles can control particle aggregation and apply the low influence of diffusion effects of lipid molecules inside the micelles and water molecules in their surroundings to adjust the relaxivities of concentrated SPIO to the static dephasing regime [15], [16]. Furthermore, the relaxivity of lipid based SPIO nanoparticles can be influenced by the exchange of bulk water with SPIO, which is dependent on the permeability of liposomal membrane to water [33]. The more permeable the membrane, the better is the water flux across the bilayer and the better the relaxivity. The volume-to-surface ratio of small lipid nanoparticle is lower and therefore the exchange with external bulk water is better [33]. In present study, we systhized a 25 nm lipid-based SPIO nanoparticle based on the static dephasing regime theory, which exhibits a constant r2* relaxivity. A linear association between the iron dose and the change in the relaxation rate (ΔR2*) in the liver 20 mins after injection of SPIO nanoparticle was observed (Fig. 2), allowing accurate quantification of blood clearance as well as SPIO uptake kinetics. The dynamic SPIO nanoparticle-enhanced MRI with two compartment modeling have showed its ability to evaluate RES function on both a cell number and receptor function level by real-time imaging blood clearance as well as hepatic uptake of SPIO nanoparticle.

Recently, a sophisticated approach to deplete macrophages has been developed based on liposome-mediated intracellular delivery of the bisphosphonate clodronate. The studies showed that macrophages in liver, spleen and extrahepatosplenic RES tissue can be irreversibly damaged by ingesting intravenously administrated clodrosome liposome [34]–[36]. Several studies on RES saturation by liposome have been reported. This strategy is based on blocking the interaction between the second dose of particles and the receptors on the macrophage cell surface and circulating serum protein by administration of a loading dose of liposome, which caused cellular receptor saturation and depletion of the serum protein, resulting in a blockaded state and an extension of lifetime of a second dose of particles in the circulation [19], [20]. It is important to note that the liposome is not toxic and can be digested in several hours after macrophage uptake; therefore liposome can't reduce number of the macrophage tissue density. In present study, we utilized the clodrosome-mediated macrophage “suicide” approach to reduce the number of macrophages in RES system and the ability of liposome on the RES saturation to evaluate the capability of the parameters derived from dynamic MRI data with the two-compartment model for investigation of RES function. After clodrosome treatment, a significant decrease in macrophage number in the livers and spleens was found, which was paralleled by the decrease in the blue dye on Prussian blue staining after SPIO nanoparticle injection. Whereas no difference in macrophage number in the livers and spleens was observed between liposome and saline group, but few of blue dye were found in liver and spleen treated with liposome compared to saline at the same dose of SPIO nanoparticles. Interestingly, a lower K_in_, a lower K_out_ and a lower K_e_ were found after clodrosome treatment, whereas a lower K_in_, a higher K_out_ and a lower K_e_ were found after liposome treatment compared to saline treatment (P<0.005, Table 3).

In our study, K_in_ and K_out_, are used to describe both passive transfer and active binding. Since particle binding to the macrophages involves two reversed processes which are association and dissociation, K_in_ (s^−1^) is used to describe the association process and dissociation is characterized by the constant K_out_ (s^−1^). The passive transfer was assumed to be same at the same dose of SPIO nanoparticles, therefore the decrease in active binding accounts for lower K_in_ and K_out_ after clodrosome treatment compared to saline treatment, indicating the decreases in SPIO nanoparticle association to and dissociation from macrophage as the number of macrophages reduced. A lower K_e_ results of a decrease in macrophage number in extrahepatic RES tissue after clodrosome treatment. Although a lower K_in_, K_out_ and K_e_ was also found in the 0.5 and 3 mg Fe/kg SPIO nanoparticle group, which is due to the decrease in passive transfer to and out of liver, since no difference in macrophage number in livers and spleens was found compared to 10 mg Fe/kg SPIO nanoparticle group. A decrease in the binding site of receptor on macrophage after liposome treatment leads to decrease in SPIO nanoparticle association to macrophage and increase in dissociation from macrophage. Thus, a lower K_in_, a higher K_out_ and a lower K_e_ were found in liposome treatment group. A lower K_e_ after liposome treatment is due to a decrease in phagocytosis function in extrahepatic RES tissue. These data indicate that the parameters K_in_, K_out_ and K_e_ derived from the dynamic data using the two-compartment model have ability to evaluate the RES function on both a cell number and recepor function level. A low K_in_ and a low K_out_ indicate a reduction of kupffer cell numbers, whereas a low K_in_ and a high K_out_ suggest the defective receptor function of kupffer cells. While, a low K_e_ indicates an impairment of extrahepatic RES function.

Several applications can be envisioned for dynamic SPIO-enhanced MRI. First, dynamic SPIO-enhanced MRI has great potential to be used as an efficient tool to investigate and stage chronic liver disease by providing the information in macrophages on a cell number and receptor function level. This would be useful for staging chronic liver injury and help for the successful individualized management of liver disease, since macrophages are integrated into all stages of inflammatory process and exhibit various functions and numbers [30], [31]. Second, dynamic SPIO-enhanced MRI might be suitable to predict the hepatic carcinogenesis, since the increased incidence of hepatocellular carcinoma complicating cirrhosis is related to the lobular and nodular depletion of liver macrophage [37]. The kinetic parameters would provide direct information on the macrophage density and activity.

## Conclusions

Dynamic SPIO-enhanced MR imaging with two-compartment modeling can provide information on RES function on both a cell number and receptor function level. This may allow more accurate diagnosis or therapy monitoring in liver disease, and may provide insight into possible therapeutic mechanisms.
