# Correction: Quantitative Evaluation of the Reticuloendothelial System Function with Dynamic MRI

**DOI:** 10.1371/journal.pone.0122323

**Published:** 2015-03-23

**Authors:** The PLOS ONE Staff

There are errors in the Funding section. The correct funding information is as follows: This study was partly supported by US Army Medical Research & Material Command Grant No. W81XWH-10–1–0604 and the National Center for Advancing Translational Sciences of the National Institutes of Health under award number UL1TR000003.

## References

[1] Liu T, Choi H, Zhou R, Chen I-W (2014) Quantitative Evaluation of the Reticuloendothelial System Function with Dynamic MRI. PLoS ONE 9(8): e103576 doi: 10.1371/journal.pone.0103576 25090653 10.1371/journal.pone.0103576 PMC4121285

